# Impact of oral mucosa lesions on the quality of life related to oral health. An etiopathogenic study

**DOI:** 10.4317/medoral.20866

**Published:** 2016-01-31

**Authors:** María-del-Carmen Villanueva-Vilchis, Patricia López-Ríos, Ixchel-Maya García, Luis-Alberto Gaitán-Cepeda

**Affiliations:** 1 Full Time Professor, National School of Superior Studies, National Autonomous University of México, Leon, Gto, México; 2 Former pregraduate student, Laboratory of Oral Pathology, Postgraduate and Research Division, Dental School, National Autonomous University of México, actually private practice dentist, México city, México; 3 Full Time Professor, Dental School, Autonomous University of Campeche, Campeche, Cam, México; 4 Chief of Laboratory of Oral Pathology, Postgraduate and Research Division, Dental School, National Autonomous University of México, México city, México

## Abstract

**Background:**

To assess the impact of oral mucosa lesions on quality of life related to oral health (QLROH) and additionally to establish whether the etiopathogenicy of oral lesion is associated to the degree of QLROH impact.

**Material and Methods:**

In this cross-sectional study performed on a non-probability sample of 247 consecutively patients attending the oral medicine and pathology clinic the Spanish version of Oral Health Impact Profile-49 questionnaire (OHIP-49-mx) was applied. Responses were recorded on Likert-type scale whose values ranged from 0 (never) to 4 (always). Values greater than the 50 percentile (median) were considered as indicative of poor quality of life. All patients were orally examined and diagnosed. In accordance to their etiopathogenicy 6 study groups were formed: 4 corresponded to MIND classification for diseases (Metabolic, Inflammatory, Neoplastic, and Development groups), with ≥2 diseases and no-lesion group. To identify possible differences of OHIP-49 values between study groups an ANOVA (one factor) parametric and a chi square tests were performed (SPSS®20.0).

**Results:**

The OHIP-49-mx values were higher than the 50 percentile (established at 39) in metabolic, inflammatory, development, and ≥2 diseases groups, suggesting that this type of oral lesions negatively impact the quality of life. ≥2 diseasesgroup followed by metabolic and inflammatory diseases group (*p* 0.001) depicted worst quality of life. Functional limitation (*p* 0.003), pain, physical inability (*p* 0.001) and psychological disabilities dimensions exhibited greater values in all groups.

**Conclusions:**

Injured oral mucosa negatively impacts quality of life, specifically functional limitation, physical inability and psychological disabilities could lead to social isolation.To our knowledge, this is the first time that an association between QLROH and the etiopathogenicy of oral mucosal diseases is established.

**Key words:**Quality of life, quality of life related to oral health, oral mucosa lesions, etiopathogenicy, MIND classification.

## Introduction

Quality of life (QL) is a multidisciplinary and multidimensional assessing concept that refers to the subjective and objective conditions related to physical, emotional and social factors (1). The World Health Organization (WHO) defines QL as “the perception of each subject on its position in life in the context of his culture and system of values in which he lives and in relation to his goals, expectations, standards and concerns” (2,3). The need to associate the perception of health with the ability to perform relevant everyday activities leads to establish the concept of quality of life related to health and consequently the quality of life related to oral health (QLROH) (2). QLROH refers to the relationship between oral circumstances associated to eating, nutrition, social interaction, emotional and psychological functions when related to discomfort, disability and social and financial impact (4). These concepts incorporate self-perception as an important component of health status assessment and promote the understanding of the way in which disease interferes with the social role of subjects (5). Due to the subjective aspect of QLROH components, quantitative indicators of functional, psychological and social effects of the disease have been developed (6) and this fact would complement the conventional dental clinical indicators used in the oral health assessment. In 1994 Slade and Spencer (7) developed the Oral Health Impact Profile-49 (OHIP-49); an instrument that assessed the frequency with which an individual experiences problems in everyday’s life functionality as a consequence of oral circumstances. OHIP-49 has been translated and validated in several countries and it is considered as a comprehensive and acceptable psychometric tool (8-10).

A relationship has been reported between QL at different ages with indicators of oral health. In children, an association has been observed between truancy, diminished study concentration and decrease in masticatory and phonation abilities, with pain caused by dental caries (11). In teenagers a sensation of inability to socialize has been reported because of halitosis (12) as well as a malocclusion-related self-esteem decline due to modifications of self-perception of personal attractiveness with respect to others (13-15). Adults with periodontitis exhibit diminished QL due to functional limitations, pain and physical disability (16-18).

The impact on QLROH has been analyzed in different socio-cultural contexts, mainly focused on, and occasionally limited to dental origin lesions, dento-facial dysmorphologies, temporomandibular joint disorders and periodontal diseases. However available scientific information is insufficient regarding the QLROH of patients with oral mucosa disease other than dental caries and periodontal disease (19,20), in spite of the fact that oral lesions and oral diseases are the most frequent causes of daily activities impairment exerting negative impact on QL. Therefore this report aims to assess the impact of oral mucosa lesions on QLROH in patients attending a teaching clinic of oral medicine; an additional aim would be to establish whether the etiopathogeny of oral lesion is associated to the degree of QLROH impact and whether oral lesions are related to any specific dimension (functional limitation, pain, psychological discomfort, physical inability, psychological inability, social inability and incapacity) of OHIP-49.

## Material and Methods

After the approbation of the protocol by the Ethical Committee from the participants Institutions, a cross-sectional study was conducted in 247 consecutively attending patients admitted at the Clinic of Medicine and Oral Pathology, from the Graduate Division, Dental School, National Autonomous University of Mexico. The period for collecting the information was from September 2009 to May 2010. Selection of individuals was conducted using a non-probability sampling, taking all the consecutive individuals that attend to receive attention during the specified period.

In accordance to inclusion criteria, all legal-age patients wishing to participate were included regardless of gender, on the other hand those patients under dental treatment or with physical or mental disability that could prevent them answering during interview were excluded. In spite of the fact that this research was considered “without risk”, all participants were requested to sign of validly informed consent form which ensured the confidentiality in the handling of data and the possibility to withdraw from the study at any moment. Thus, each subject referred for first time to the oral medicine and oral pathology clinic was invited to participate in the research, and had received explanations of the purpose of the study, its scope and procedure to follow. At that moment a questionnaire concerning demographic data was conducted. At a later point in time the Oral Health Impact Profile questionnaire, consisting of 49 items, in its Spanish version (OHIP-49-mx) was applied. The Spanish version of OHIP-49 had been previously validated (21).

OHIP is a tool whose main objective is to quantify the frequency with which the patients face difficulties to carry out daily activities because of oral diseases. OHIP-49-mx has 49 items representing seven dimensions: functional limitation, pain, psychological disability, physical inability, psychological distress, social inability and inability to function (incapacity). The responses were recorded on a Likert-type scale whose values ranged from 0 (never) to 4 (always). The final score ranged from 0 to 196 and was calculated using the additive method of responses recorded by reactive. Possible scores on each dimension values were: from 0 to 36 for functional limitation, pain and physical disability; from 0 to 24 for psychological distress and inability to function, from 0 to 20 for psychological disability and social inability. The 50 percentile (median) was used as a cutoff point to determine the negative impaction the quality of life. Thus, those values greater than the 50 percentile scores (median) were considered as reflecting poor quality of life.

As part of their clinical protocol, all patients were comprehensively examined by an expert in medicine and oral pathology using dental mirrors under artificial light. If necessary, clinical diagnoses were histopathologically corroborated. With research purposes in mind all patients were grouped in 6 study groups: 4 corresponded to MIND classification for diseases (Metabolic, Inflammatory, Neoplastic, and Development groups) (22,23), and two additionally groups were included: those with two or more concomitantly diseases, and patients without clinical evidence of oral lesion or oral disease (no-lesion group).

A descriptive analysis of socio-demographic variables and use of alcohol and tobacco was conducted. To identify possible differences between mean OHIP-49 and of each dimension of OHIP-49-mx an ANOVA (one factor) parametric test and a chi square test (*p* <0.05 IC95%) were performed. In both situations the software program SPSS®20.0 was used.

## Results

247 patients (179 female; 68 male) were included. According to MIND classification 5 (2.07%) of patients were diagnosed with metabolic diseases; 139 (56.3%) suffered some inflammatory disease or lesion; 13 (5.3%) were diagnosed with neoplasia (benign or malignant) and 15 (6%) suffered developmental diseases. 7 patients (2.8%) of all patients were afflicted with 2 or more different oral diseases/lesions and 27.5% of total patients included did not exhibit evidence of oral mucosa lesions or oral disease affecting oral mucosa. Total data are presented in  Table 1.

**Table 1:**
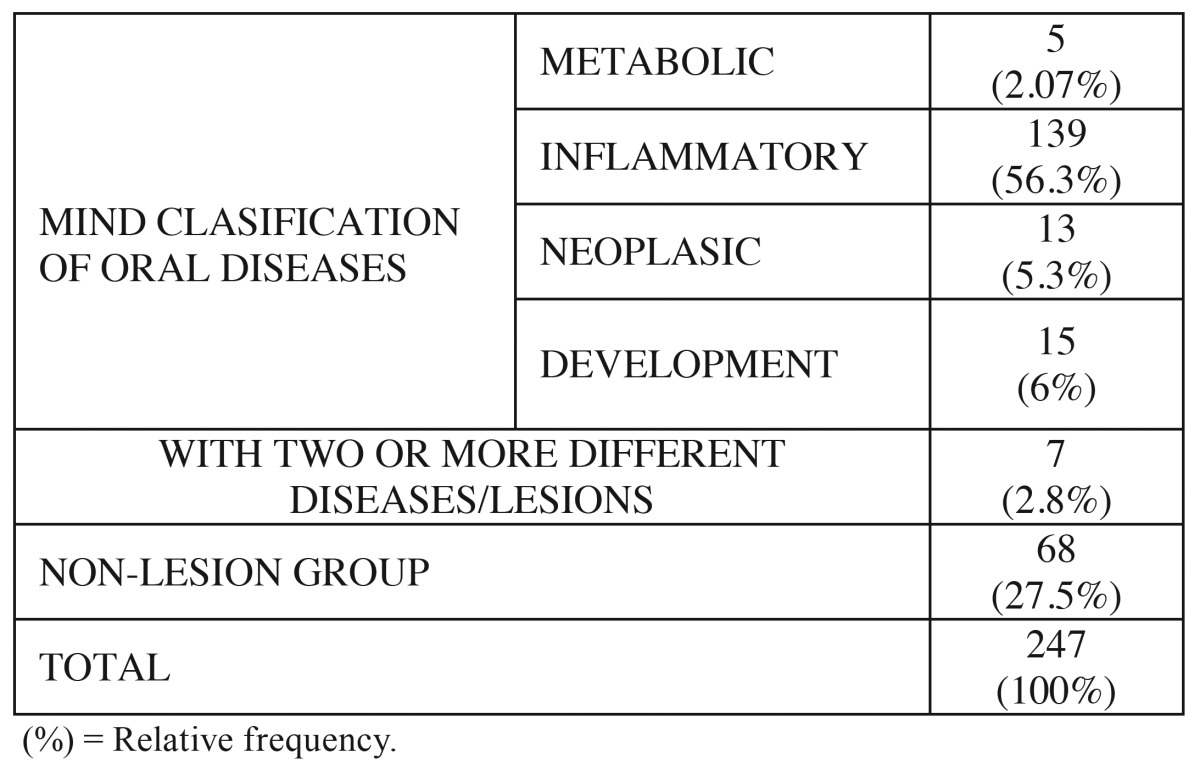
Distribution of patients with respect to etiopathogenicy (mind clasification).

The total value of OHIP-49-mx and the values per dimension are shown in  Table 2. The mean of OHIP-49-mx obtained in all study groups was higher than the 50 percentile (established at 39). These data suggest that oral lesions, regardless of their etiopathogenicy, negatively impact patient’s QL. Our results showed that the ≥2 oral diseases/lesions group possesses the greater difference with respect to cutting point (72.2 vs. 39 respectively). This association was statistically significant (*p* 0.001). With respect to MIND classification, metabolic disease group exhibited highest value 63 (±55) and all dimensions of OHIP-49-mx were above the cutting-off point and thus produced negatively impact on the quality of life of patients suffering them .

**Table 2:**
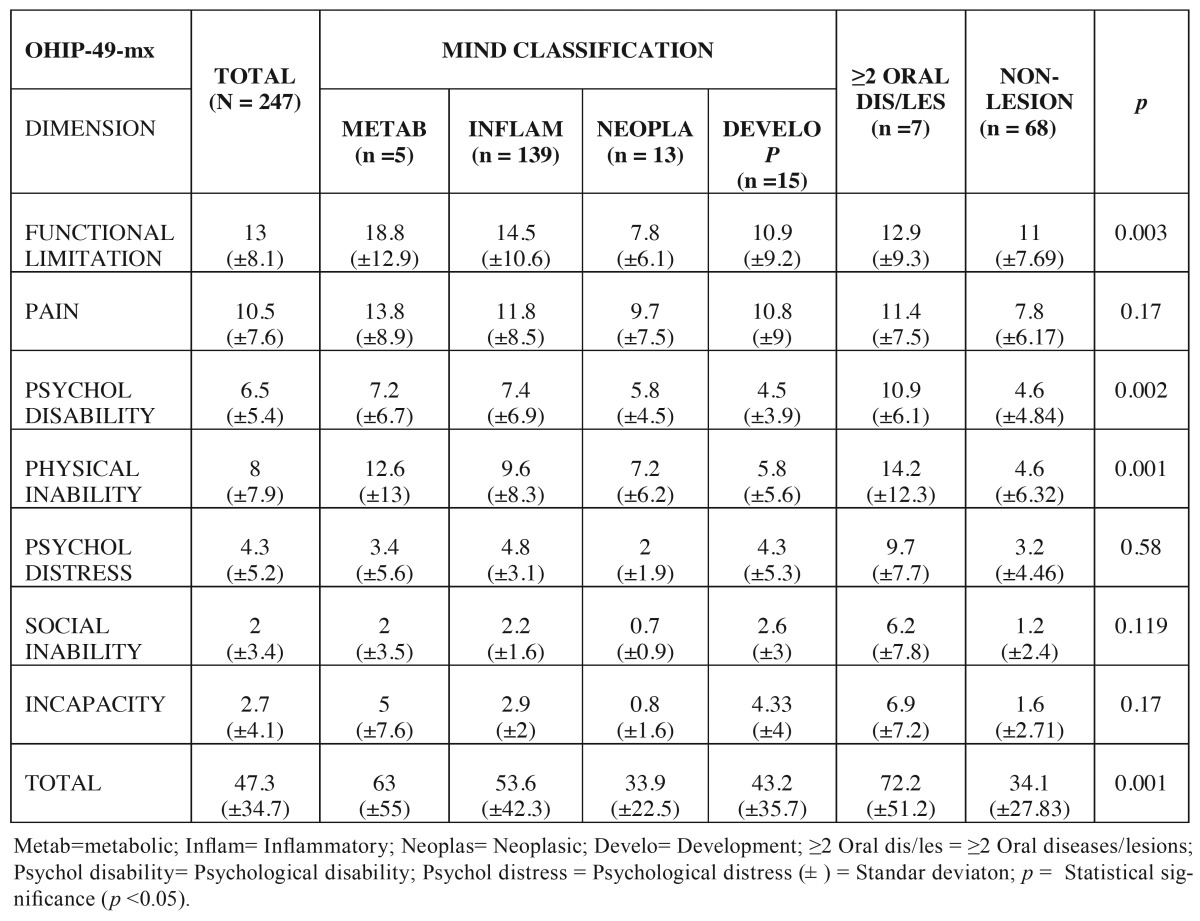
Average values of ohip-49mx dimensions with respect to etiopathogenicy of oral diseases.

With respect to the dimensions of OHIP-49-mx, we found that functional limitation (*p* 0.003), pain, physical inability (*p* 0.001) and psychological disability (*p* 0.002) dimensions exhibited greater values in the total of sample as well as in all study groups ( Table 2). The statistical analysis shows a significant association of functional limitation and metabolic disease group (*p* 0.003); and of psychological disability (*p* 0.002) and physical inability (*p* 0.001) with patients suffering ≥2 oral diseases/lesions concomitantly.

With respect to type of disease, the highest relative frequency of patient with scores higher than the median corresponded to the ≥2 oral diseases/lesions group followed by metabolic and inflammatory diseases groups. The association of poor QL and inflammatory diseases were statistical significant (*p* 0.005). Patients suffering inflammatory oral lesions experience a 20.5 fold risk of experiencing a poor quality of life (Odds ratio 20.5 IC 1.19 - 3.53) than other study groups. On the other hand 66.2% of subjects belonging to the no-lesion group reported good QLROH.  Table 3 shows the distribution of relative frequencies of patients with poor QL in relation to the etiopathogenicy of disease.

**Table 3:**
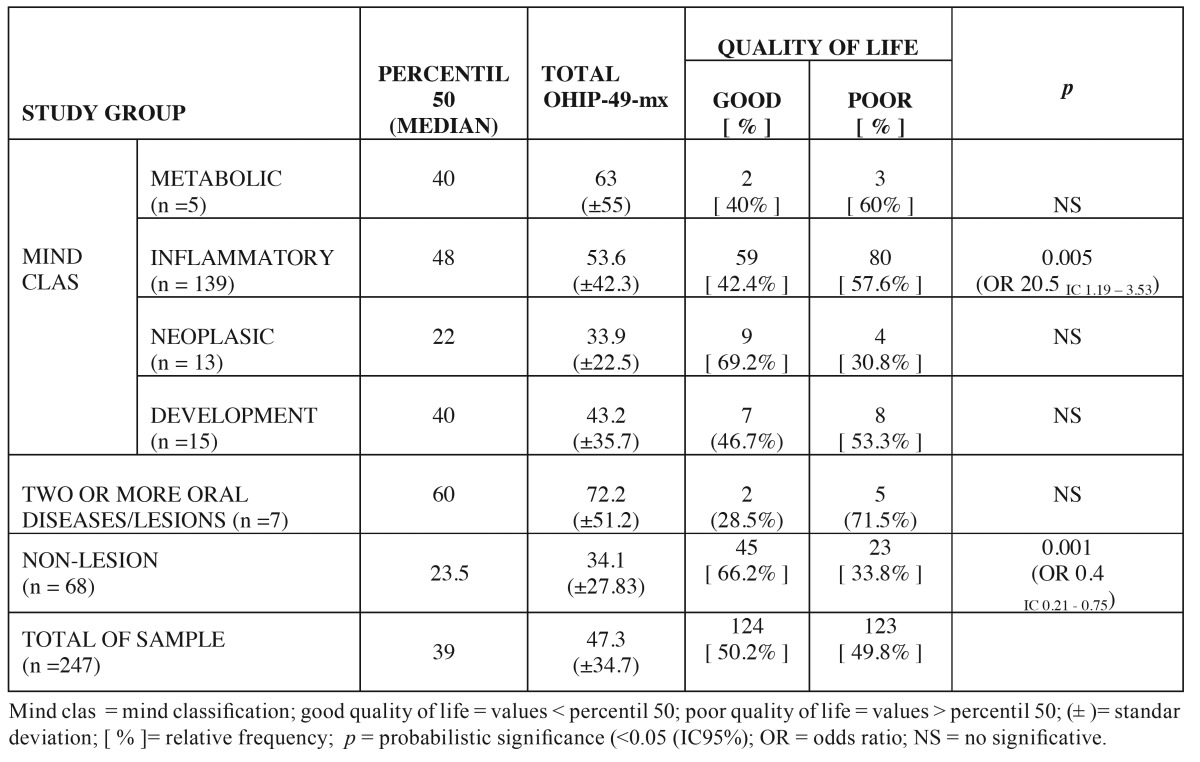
Relative frequency of patients with good or poor quality of life with respect to etiopathogenicy of oral lesions.

## Discussion

This report shows that oral mucosa lesions negatively impact the quality of life of patients suffering them. This result is in agreement with those obtained in other populations. In an Arabian population it was reported that mucocutaneous diseases with oral mucosa manifestations negatively impacted QLROH and that the degree of impact was directly related to the amount of oral lesions (20). This last issue could not be compared with results obtained in the present study because lesions were not specifically recorded. On the other hand these authors identified the fact that eating was the most frequently everyday activity which resulted impacted as well as the pain associated to mucosal lesion (20). Our results showed that pain dimension presented highest score on OHIP-49-mx.

To our knowledge, this is the first time that a correlation of QLROL to etiopathogenicy of oral mucosal diseases is established. OHIP-49-mx is an instrument primarily designed to assess QLROH related to dental circumstances and information of their use with respect to oral mucosa lesions is scarce. OHIP-49-mx covers all the major domains of QLROH and can therefore also be used in lesions of the oral mucosa. This report addressed the question of whether the etiopathogenicy influences or not QL degree of deterioration. Patients involved in the present research were grouped in 6 categories, 4 corresponding MIND classification (metabolic, inflammatory, neoplasm and development groups) (22,23) and two groups were added: with 2 or more concomitantly different diseases and the second group was formed by patients who did not exhibit oral mucosa lesions. Our results showed that subjects with ≥2 oral diseases/lesions and those suffering metabolic diseases presented poorest QL. In the case of ≥2 oral diseases/lesions group this result could be consequence of additive effect of several diseases at one time, while the association of metabolic diseases and poor QL could be due to the fact that the 5 subjects had been diagnosed with type II diabetes. It is known that the diabetic peripheral neuropathy, including orofacial pain, is one of the most common complications of long evolution for the diabetic patient, affecting approximately 50% of this population (24). However the small sample of metabolic group (n = 5), did not allow to generalize these data and therefore they must be taken with caution and should count with corroboration of other study groups.

The inflammatory group was the largest one. This study group possessed the third highest score suggesting it exerted a strong negative impact on QLROH. This study group included vesicular disease. Vesicular diseases have been closely associated to poor QL. The frequency of patients with poor QLROH who suffer from vesicular oral diseases is higher than 77% (25). Previous studies mentioned that symptomatic oral lichen planus patients have a poorer QL than patients affected by asymptomatic oral lichen planus (20). The same situation has been reported in subjects that suffer ulcers, erosion and symptomatic oral lesions compared to their counterparts with asymptomatic lesions (25). Patients suffering oral mucosa lesions which radically affect QLROH, e.g. patients with recurrent aphthous stomatitis, showed a very high frequency of psychological problems (26). Our results show high values in psychological disability dimension in all study groups, specifically in inflammatory disease group.

A necessity arises to clarify the case of burning mouth syndrome. The burning mouth syndrome is characterized by a burning sensation or pain in the oral mucosa, lacking clinical or morphological signs of disease, or medical or dental identifiable causes. Therefore it has been suggested as being a symptom of neuropathic pain [for a review see Silvestre *et al*. (27). A very strong negative impact on QLROH of subjects who suffer burning mouth syndrome has been reported. Patients with burning mouth syndrome always showed worst the scores regardless of the tool to measure QL (19,28).The present study did not include patients with burning mouth syndrome because they did not show any morphological or physical changes or elementary lesions of oral mucosa and therefore could not be included into MIND classification. This fact undoubtedly constitutes a weakness in the present paper.

The inclusion of a study group formed by subjects without oral lesion in a study of the impact of oral lesions on QL could be considered paradoxical. It is necessary to point out that all patients attending the teaching dental clinic of the Dental School, National Autonomous University of Mexico, and who exhibit changes in their oral mucosa are referred to the oral medicine clinic for confirmation or dismissal of diagnosis. In such way there are subjects examined at the oral medicine clinic that do not present lesions or disease in the oral mucosa. Surprisingly this study group did not exhibit the lower score of OHIP-49-mx, and except for pain and physical disability, did not present the lower values by dimensions. The non-lesion group score of OHIP-49-mx were very similar to those found in the neoplasic group, 34.1 vs 33.9 respectively. Even more, 33.8% of the subjects of the non-lesion group suffered poor quality of life, while the 30.8% of subjects belonging to neoplastic lesions group exhibited poor QLROH. The negative impact on QLROH observed in the non-lesion group could be related to dental illness such as tooth decay, toothache or to the functionality of dental prostheses. These dental needs in accordance to clinical protocol of our institution were not diagnosed or treated in the oral medicine clinic. However, we cannot exclude the presence of dental complications in the non-lesion group and therefore justify the scores of OHIP-49-mx. Values obtained in the non-lesion study group were similar to those obtained in patients with neoplasm. This data give rise to several considerations: 1) A limitations of MIND classification is that it does not specify the biological behavior of neoplasia (malignant or benign), and although assessing the prevalence or relative frequency of benign and malignant neoplasms is beyond the aim of this paper, clinical experience suggests that the number of patients with a diagnosis of benign neoplasm is far greater than those diagnosed with malignant neoplasm; 2) it is known that benign neoplasms are asymptomatic and produce little pain and discomfort which are generally associated to local complications; 3) the majority of malignant neoplasms are not symptomatic in early stages. The fact that neoplasms do not show high values in pain dimension, suggest there is no clinical alert associated to the presence of those neoplasmsand in consequence oral examination by the clinician assumes enormous importance in order to make an early diagnosis.

The present report found that physical and social disabilities dimensions were affected in all types of oral mucosa lesions. This could be interpreted as the fact that injured oral mucosa regardless of its etiopathogenicy negatively impacts the QLROH and could lead to social isolation. A specific condition results in a deficiency; which in turn produces a disability and a handicap in the subject, therefore it customizes everyday activities on a regular basis. In this respect, this report demonstrates that patients with lesions of the oral mucosa presented poor QLROH and that oral mucosa lesions bear influence in the deterioration of their everyday activities in addition to involving the context in which they take place. Oral diseases and tissue damage are predictors of poorer quality of life. Therefore, a healthy mouth will contribute to the subject’s well-being and will contribute to his personal satisfaction and happiness.
